# In Mice, Tuberculosis Progression Is Associated with Intensive Inflammatory Response and the Accumulation of Gr-1^dim^ Cells in the Lungs

**DOI:** 10.1371/journal.pone.0010469

**Published:** 2010-05-04

**Authors:** Irina V. Lyadova, Evgeny N. Tsiganov, Marina A. Kapina, Galena S. Shepelkova, Vasily V. Sosunov, Tatiana V. Radaeva, Konstantin B. Majorov, Natalya S. Shmitova, Henk-Jan van den Ham, Vitaly V. Ganusov, Rob J. De Boer, Rachael Racine, Gary M. Winslow

**Affiliations:** 1 Department of Immunology, Central Tuberculosis Research Institute, Russian Academy of Medical Sciences, Moscow, Russian Federation; 2 Department of Theoretical Biology and Bioinformatics, Utrecht University, Utrecht, The Netherlands; 3 Division of Infectious Diseases, Wadsworth Center, New York State Department of Health, Albany, New York, United States of America; 4 Department of Post-Graduate Studies, Institute of Biophysics SB RAS (Russian Academy of Sciences, Siberian Branch), Krasnoyarsk, Russian Federation; New York University, United States of America

## Abstract

**Background:**

Infection with *Mycobacterium tuberculosis* (*Mtb*) results in different clinical outcomes ranging from asymptomatic containment to rapidly progressing tuberculosis (TB). The mechanisms controlling TB progression in immunologically-competent hosts remain unclear.

**Methodology/Principal Findings:**

To address these mechanisms, we analyzed TB progression in a panel of genetically heterogeneous (A/SnxI/St) F2 mice, originating from TB-highly-susceptible I/St and more resistant A/Sn mice. In F2 mice the rates of TB progression differed. In mice that did not reach terminal stage of infection, TB progression did not correlate with lung *Mtb* loads. Nor was TB progression correlated with lung expression of factors involved in antibacterial immunity, such as iNOS, IFN-γ, or IL-12p40. The major characteristics of progressing TB was high lung expression of the inflammation-related factors IL-1β, IL-6, IL-11 (p<0.0003); CCL3, CCL4, CXCL2 (p<0.002); MMP-8 (p<0.0001). The major predictors of TB progression were high expressions of IL-1β and IL-11. TNF-α had both protective and harmful effects. Factors associated with TB progression were expressed mainly by macrophages (F4-80^+^ cells) and granulocytes (Gr-1^hi^/Ly-6G^hi^ cells). Macrophages and granulocytes from I/St and A/Sn parental strains exhibited intrinsic differences in the expression of inflammatory factors, suggesting that genetically determined peculiarities of phagocytes transcriptional response could account for the peculiarities of gene expression in the infected lungs. Another characteristic feature of progressing TB was the accumulation in the infected lungs of Gr-1^dim^ cells that could contribute to TB progression.

**Conclusions/Significance:**

In a population of immunocompetent hosts, the outcome of TB depends on quantitatively- and genetically-controlled differences in the intensity of inflammatory responses, rather than being a direct consequence of mycobacterial colonization. Local accumulation of Gr-1^dim^ cells is a newly identified feature of progressing TB. High expression of IL-1β and IL-11 are potential risk factors for TB progression and possible targets for TB immunomodulation.

## Introduction

Approximately one third of the human population is infected with *Mtb*. The majority of infected individuals remain free from active disease for life [1]. Approximately 10% of infected individuals progress to clinical TB. The disease characteristics exhibited by this population are very diverse, and differ by the type of pathology developed in the lungs, the area of affected lung tissue, the presence of acid fast bacilli in the sputa, and the rate of TB progression. While in most cases TB progresses slowly, several forms of disease, e.g., caseous pneumonia, disseminated TB, progress rapidly. Mechanisms that determine the outcome of *Mtb* infection and the rate of TB progression remain largely unknown.

Analysis of the immunological mechanisms involved in the control of TB in humans is complicated, due to a number of factors, including differences in the exposure to mycobacteria, virulence of infecting *Mtb* strains, variability in host genetic and socio-economic factors [2]. In contrast, mouse models are well-controlled experimental tools to address TB immunity [3]–[7]. Studies in gene-targeted mice have identified several cell subsets (e.g., CD4 and CD8 T cells) and molecules (e.g., IFN-γ, TNF-α) whose deficiency results in extremely severe TB and suggested that active TB develops as a result of inefficient antibacterial responses [4]–[7]. This scenario explains why hosts with genetic or acquired deficiencies in their antibacterial immune response suffer from severe mycobacterial infections. However it does not explain why active TB occurs in immunologically-competent hosts, nor why TB exhibits so many different clinical manifestations. These questions are not easily addressed by gene targeting or neutralization/depletion experiments. Indeed, a complete absence of a particular gene is a rare situation in a human population. In addition, the majority of cells and molecules mediating immune response play multiple (i.e., protective and pathological) roles in TB pathogenesis of which only one can be discerned by gene targeting approach.

Thus, an alternate experimental approach has been used to address TB immunity that involves the comparison of host responses in mouse strains with different susceptibilities to infection (e.g., C57BL/6 *versus* DBA/2; C3HeB/FeJ *versus* C57BL/6; I/St *versus* A/Sn). This approach has identified a number of differences in innate and acquired immunity between susceptible and resistant strains [8]–[18]. In most cases, however, it is difficult to judge whether the identified inter-strain differences contribute to protection or pathology, as most mouse strains carry combinations of resistance and susceptibility genes and their phenotypes may represent a mixture of both protective and pathological responses.

In previous studies, we described differences in TB severity between two strains, I/St and A/Sn. Compared to A/Sn mice, infection of I/St mice with *Mtb* resulted in higher mycobacterial loads, more severe lung tissue pathology, and earlier morbidity [10], [11], [14], [16]. Immunological analysis showed that I/St mice differed from less susceptible A/Sn mice in that they exhibited lower antimycobacterial responses [11], [17] and more prominent inflammatory reactions [11], [16], [18]. The relative impact of these responses in the protection against (progression of) TB remained unknown.

To elucidate whether and how the identified differences between the I/St and A/Sn strains contribute to disease control, in the present study we analyzed TB severity and lung immune responses in a panel of genetically heterogeneous F2 progeny of I/St and A/Sn strains. This approach allowed us to use a “natural” genetically unmodified population, and to directly associate the inter-strain differences identified in I/St and A/Sn mice with TB protection or pathology. We report that in F2 mice the major characteristics of progressing TB was not the number of mycobacteria growing in the lung, or a deficiency in factors supporting antibacterial immunity. Rather, TB progression was associated with high inflammatory response mounted by host phagocytic cells and manifested as high lung expression of the inflammation-related factors IL-1β, IL-11, CCL3, CXCL2, MMP-8, and a progressive accumulation of Gr-1^dim^ cells in the lungs.

## Results

### F2 mice display different rates of TB progression that in most mice do not correlate with mycobacterial load

**Figure 1:**
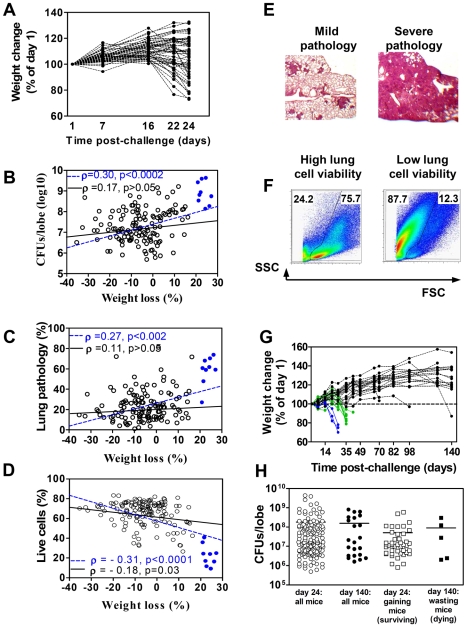
F2 mice display different rates of TB progression. F2 mice were challenged i.t. with 10^3^ CFU of *Mtb*. Weight was monitored once a week. A, Kinetics of weight change. 100% - weight on day 1 post-infection. Shown are representative results obtained in two (n = 52) independent experiments that included mice of both sexes. B–D, Mycobacterial load (B), lung pathology (C), and lung cell viability (D) in mice displaying different degree of wasting. Severely wasting mice (wasting by more than 20%) are indicated by blue circles. Lines show the predictions of the linear regression when all mice are included in the analysis (dashed blue lines) or when severely wasting mice are excluded from the analysis (solid black lines). ρ, Spearman correlation coefficient; p, p-value for ANOVA. E, F, Examples of lung tissue sections and lung cell flow cytometry representing two extremes with mild and severe pathology. Numbers in F indicate the percentages of dead (left) and live (right) cells. G, H, Long-term monitoring of F2 mice (n = 30, two independent experiments). G, The kinetics of weight change. H, Comparison of mycobacterial loads observed on days 24 and 140 post-infection.

In the first set of experiments we analyzed the kinetics of TB progression and addressed the correlation between TB progression, mycobacterial multiplication, and lung tissue pathology in (A/SnxI/St) F2 mice. F2 mice originated from TB-highly-susceptible I/St and more resistant A/Sn mice. The mice were infected with *Mtb*, and TB progression was monitored by evaluating post-infection body weight loss, a vital indicator of TB severity in experimental animals and humans (Figure 1). During the first 2 weeks of infection, all mice gained weight. At the end of week 3, some mice started to undergo wasting. On day 24, the mice displayed a great variability in the degree of wasting (Figure 1A). At this time, lungs were isolated from individual mice and used for: (i) determination of mycobacterial load, (ii) examination of lung tissue pathology, (iii) flow cytometry analysis, and (iv) gene expression analysis.

Microbiological, histological, and flow cytometry examinations revealed that all mice displayed a range of *Mtb* loads, lung pathology, and lung cell viability (Figure 1 B–F). We found a direct correlation between wasting and mycobacterial load (Figure 1B, blue line), and between wasting and lung tissue pathology (Figure 1C, blue line), and an inverse correlation between wasting and lung cell viability (Figure 1D, blue line). However, the major contributors to these correlations were mice that by day 24 had lost more than 20% of their initial weight (i.e., “severely wasting” mice): these mice had high *Mtb* loads, severe lung pathology and low lung cell viability (Figures 1 B–D, blue circles). Hierarchical clustering analysis revealed that these mice were outliers and formed a separate group (File S1, Figure S1).

In two separate experiments, we performed long-term monitoring of the infected F2 mice. We found that mice that on day 24 wasted by more than 20% died within 1–3 days (Figure 1G, blue lines), mice that exhibited lower degree of wasting (<20%, “moderately wasting” mice) survived for additional 7–12 days (Figure 1G, green lines), and the majority of gaining mice survived for as long as 140 days post-infection (i.e., the time at which some of them started to waste and all mice were sacrificed for the analysis, see below). These results indicated that processes occurring during first 3–4 weeks of infection determined the outcome of *Mtb* infection and revealed that mice severely wasting on day 24 were already terminally ill.

Because terminal stage of infection may dramatically change disease manifestations (e.g., cause a secondary loss of *Mtb* control, as in the study of Yan and coauthors [19]), we next removed severely wasting mice from the analysis, and addressed the correlations between wasting, mycobacterial load, and lung pathology in all other mice. This approach made the correlations between wasting and lung *Mtb* loads and wasting and lung tissue pathology statistically non-significant (Figure 1 B, C solid lines).

Thus, only in some (i.e., terminally ill) mice, TB progression was associated with high mycobacterial loads. In the majority of mice the rate of TB progression did not significantly depend on bacterial load.

To further address an association between TB progression and mycobacterial multiplication, we performed microbiological examination of surviving F2 mice on day 140 post-infection. We found that long-lived mice had mycobacterial loads in the same range that the gaining and moderately-wasting mice had on day 24 post-infection (Figure 1H). In particular, no differences in mycobacterial loads were detected between mice that gained weight on day 24 (i.e., those that would survive for several months), and mice that exhibited wasting on day 140 (i.e., were dying). These data further suggested that in the majority of F2 mice the disease outcome was not a direct consequence of the number of mycobacteria growing in the lungs.

### Only one of the three QTLs that control TB progression in F2 mice is involved in the control of Mtb colonization

Earlier genetic analyses identified three major quantitative trait loci (QTLs) that influenced post-infectious body weight loss in I/St and A/Sn mice: *tbs1* located on chromosome 3, *tbs2* located on chromosome 9, and a QTL located in the vicinity of H-2 complex on chromosome 17 [8], [14]. In the current study, we examined whether these QTLs were involved in the control of mycobacterial colonization in F2 mice. We found that only QTL located on chromosome 17 was associated with the number of *Mtb* growing in the lungs (LRS = 11.3, Table 1). This QTL also controlled the number of IFN-γ producing CD4^+^ T-cells in the lungs (LRS = 11.4, Table 1), indicating that chromosome 17 QTL influenced *Mtb* colonization by controlling host antibacterial immune response. *Tbs 1* and *tbs2* did not show significant association with mycobacterial colonization in F2 mice (LRS<6, p>0.01), supporting our previous conclusion that the number of mycobacteria growing in the lungs was not the sole factor that determined disease outcome in F2 mice and that there were other factors that drove disease progression.

**Table 1:** Only one of the three quantitative trait loci (QTLs) controlling TB progression in F2 mice, is involved in the control of mycobacterial colonization.

Trait	Marker	LRS	P	% variance explained	CI	Mode
*Mtb* colonization CFUs/lung)	D17Mit175	11.3	0.00079*	12	50	Recessive
	D3Mit299	3.7	0.055	4	147	Recessive
	D9Mit89	0.1	0.74	0	4841	Recessive
CD4^+^ IFN-γ^+^ cells (%)	D17Mit175	11.4	0.00074*	11	49	Additive
	D3Mit299	5.2	0.0225	5	105	Recessive
	D9Mit89	1.7	0.195	2	318	Additive

F2 female mice (n = 92) challenged with *Mtb* were analyzed for mycobacterial load and the frequency of IFN-γ producing T cells in the lungs. LRS, likelihood ratio statistic, CI, a 95% confidence interval.

*Less than empirical significance level for 1000 permutations.

### TB progression correlates with increased lung expression of inflammation-related factors

Searching for factors that could play a role in TB progression in F2 mice, we focused our attention on our previous data that detected differences between parental I/St and A/Sn mice with respect to the intensity of lung tissue inflammation. In particular, I/St mice characteristically exhibited an extensive infiltration of the infected lungs with T-lymphocytes and granulocytes (Gr-1^+^ cells [18]). Because the accumulation of immune cells at the site of infection is orchestrated by cytokines and chemokines, we decided to evaluate cytokine and chemokine expression in the lungs of mice with different susceptibility to TB.

**Figure 2:**
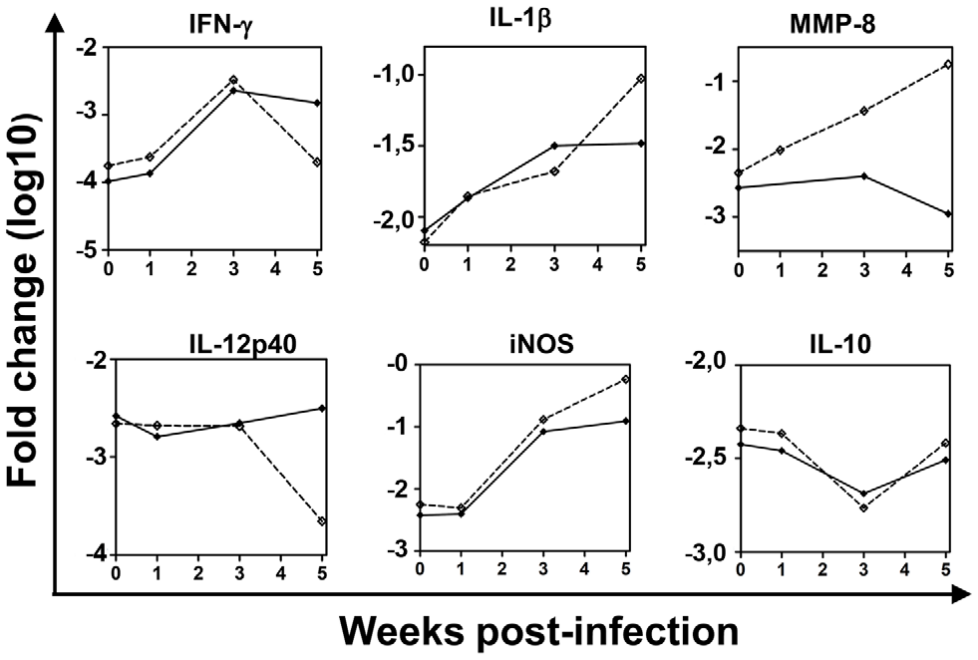
TB-susceptible I/St and TB-resistant A/Sn mice infected with Mtb differ by the expression of genes associated with T-cell mediated response and inflammation. I/St and A/Sn mice were challenged with *Mtb* or left un-infected. The expression of genes was analyzed in the lungs at weeks 0, 1, 3, and 5 post-challenge. Shown are typical examples of the expression of genes which were down-regulated in I/St lungs at late time-points post-challenge (IFN-γ, IL-12p40), up-regulated in I/St lungs at late time-points post-challenge (IL-1β, iNOS), up-regulated in I/St lungs at all time-points post-infection (MMP-8), and genes that were expressed similarly in I/St and A/Sn lungs (IL-10). Mean ±SD are shown (n = 3–4 per time-point). I/St, dashed line; A/Sn, solid line.

The first set of experiments was performed in I/St and A/Sn mice, and addressed the expression of factors involved in the antibacterial immune response (i.e., IFN-γ, TNF-α, IL-10, IL-12p40, T-bet, iNOS), inflammation (IL-1β, IL-6, TNF-α, CCL2, CCL5, CXCL2, iNOS), and factors that previously had been shown to be differentially expressed in I/St and A/Sn mice (IL-6, IL-11, MMP-8, MMP-10 [18], [20]). Mice were challenged with *Mtb*, and gene expression in the lung tissue was analyzed using quantitative PCR at weeks 0, 1, 3, and 5 post-challenge. Until the third week post-infection, the expression of the majority of genes analyzed were similar in mice of both strains. By week five, the expression of genes associated with T cell-mediated antibacterial immune response (IL-12p40, IFN-γ, T-bet, CCL5) declined in I/St mice, resulting in a relative deficiency of these factors in the lungs of susceptible mice (Figure 2 and data not shown). In contrast, the expression of inflammation-related genes (IL-1β, IL-6, TNF-α, CXCL2) and iNOS increased in I/St lungs, resulting in a relative abundance of these factors in the lungs of susceptible mice. The expression of IL-10 and CCL2 did not differ significantly between the mice at any time-point. In contrast, IL-11, MMP-8, and MMP-10 were over expressed in I/St mice at all analyzed time-points (Figure 2 and data not shown).

Based on these results, we chose a set of factors to be evaluated in F2 mice. This set included the cytokines IFN-γ, IL-12p40, IL-1β, IL-6, TNF-α, IL-11; the chemokine CXCL2; iNOS, MMP-8 and MMP-10. In addition, CCL3 and CCL4, chemokines known to mediate T lymphocyte chemotaxis were included in the analyses. The analyses was performed on day 24 post-infection, a time when all F2 mice remained alive, and disease manifestations in the acute- and chronically-infected mice diverged. The expression of each of the factors was correlated with TB progression using Spearman correlation analysis. To exclude any influence of gender on the results, gene expression analysis was performed only in females.

**Figure 3:**
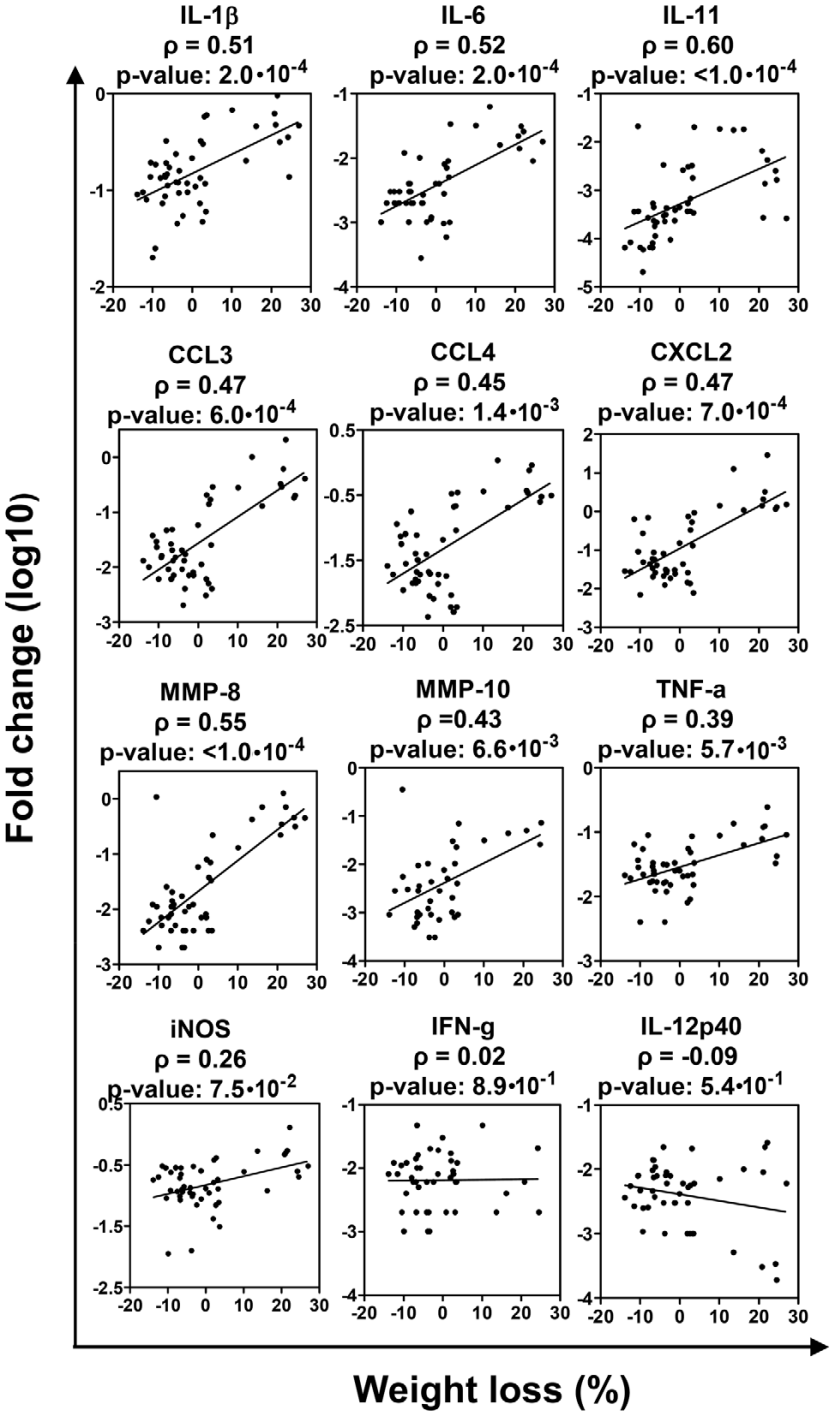
In F2 mice, TB progression correlates directly with lung expression of pro-inflammatory cytokines, chemokines, and metalloproteinases. F2 mice were challenged with *Mtb* as described in Figure 1. On day 24, lung mRNA was extracted and assayed by real-time PCR. Shown are the correlations between wasting and lung mRNA expression of indicated genes in F2 female mice (n = 48).

Gene expression was first analyzed in 48 mice. We found a strong positive correlation between TB progression and lung expression of the inflammation-related factors IL-β, IL-6, IL-11, CCL3, CCL4, CXCL2, and MMP-8 (Figure 3, p<0.001). The expression of TNF-α and MMP-10 correlated weakly with TB progression, and was not significant for multiple parameter testing (p = 0.0057 and 0.0066, respectively; to account for testing of 12 parameters, p-value of 0.003 was considered significant). The expression of iNOS, IFN-γ, and IL-12p40 did not show correlation with TB progression (p>0.07).

To better address an association between TB progression and inflammatory reaction in the lungs, we selected several factors (IL-1β, IL-11, TNF-α, CCL3, CXCL2, MMP-8 and iNOS) and evaluated their expression in an additional 27 mice. The results obtained in all 75 mice were then analyzed using different methods of correlation analysis.

Simple correlation analysis of weight loss and a single factor revealed a correlation between TB progression and lung expression of IL-1β, IL-11, CCL3, CXCL2, and MMP-8. Among these factors, IL-1β, IL-11, and MMP-8 correlated the best with the weight loss (p<0.0003, Table 2, “All”).

**Table 2:** Correlation between weight loss, mycobacterial load, and the expression of inflammation-related factors in the lungs of F2 mice.

Variable	ρ	All	Gaining+moderately wasting (n = 68)
	p-value	(n = 75)	
Mycobacterial load	ρ	**0.33**	0.12
	p-value	**3.8×10^−3^**	3.1×10^−1^
IL-1β	ρ	**0.51**	**0.40**
	p-value	**<1.0×10^−4^**	**6.0×10^−4^**
IL-11	ρ	**0.42**	**0.41**
	p-value	**2.0×10^−4^**	**5.0×10^−4^**
TNF-α	ρ	0.2	0.1
	p-value	8.0×10^−2^	6.7×10^−1^
CCL3	ρ	**0.41**	0.24
	p-value	**3.0×10^−4^**	5.2×10^−2^
CXCL2	ρ	**0.36**	0.18
	p-value	**1.3×10^−3^**	1.6×10^−1^
MMP-8	ρ	**0.46**	0.30
	p-value	**<1.0×10^−4^**	1.0×10^−2^
iNOS	ρ	0.20	0.04
	p-value	8.5×10^−2^	7.0×10^−1^

F2 female mice were challenged with *Mtb* and analyzed on day 24 post-infection (n = 75). Correlations between wasting, *Mtb* loads and indicated gene expression were analyzed in two different ways: (i) all mice were included in the analysis (“all”); (ii) severely wasting mice were removed from the analysis (“gaining+moderately wasting”). ρ, Spearman coefficient, p-value, significant value of the test. Correlations significant for multiple (eight) parameter testing are shown in bold (p<0.006). Correlations with p-value>0.006 but less than 0.05 are marked with an asterisk.

Because severely wasting (terminally-infected) mice formed a separate group (File S1, Figure S1), we next removed them from the analysis, and examined which factors contributed to the variation in the rate of TB progression in other mice. Using simple correlation analysis, we found that IL-1β and IL-11 were best correlated with weight loss in gaining and moderately wasting mice (p≤0.0006, Table 2). A weaker correlation was observed between TB progression and lung expression of MMP-8 (p = 0.01, Table 2). Other approaches, such as F-test for nested linear models, multiple regression model selection, using Akaike or Bayesian Information Criterion, bootstrapping analysis, also indicated IL-11 and/or IL-1β as the best predictor(s) of TB progression (i.e., weight loss) in gaining and moderately wasting F2 mice (Files S2, S3, S4, Table S1). These results contrasted with the absence of correlation between TB progression and *Mtb* burden in non-terminally ill mice (Table 2), and suggested a possible role for the above factors in TB progression at early (pre-terminal) stages of disease.

The correlation between TB progression and lung expression of TNF-α was different, depending on the analysis performed. In simple correlation analysis, there was a weak direct correlation between TB progression and the expression of TNF-α. However, when other factors, especially IL-1β and IL-11, were taken into account, TNF-α was correlated negatively with TB progression, exhibiting a small protective effect (File S4, Table S1).

To summarize, the high expression of inflammation-related factors was the most characteristic indicator of severe TB in F2 mice.

### The extent of host inflammatory response does not directly depend on Mtb colonization

**Figure 4:**
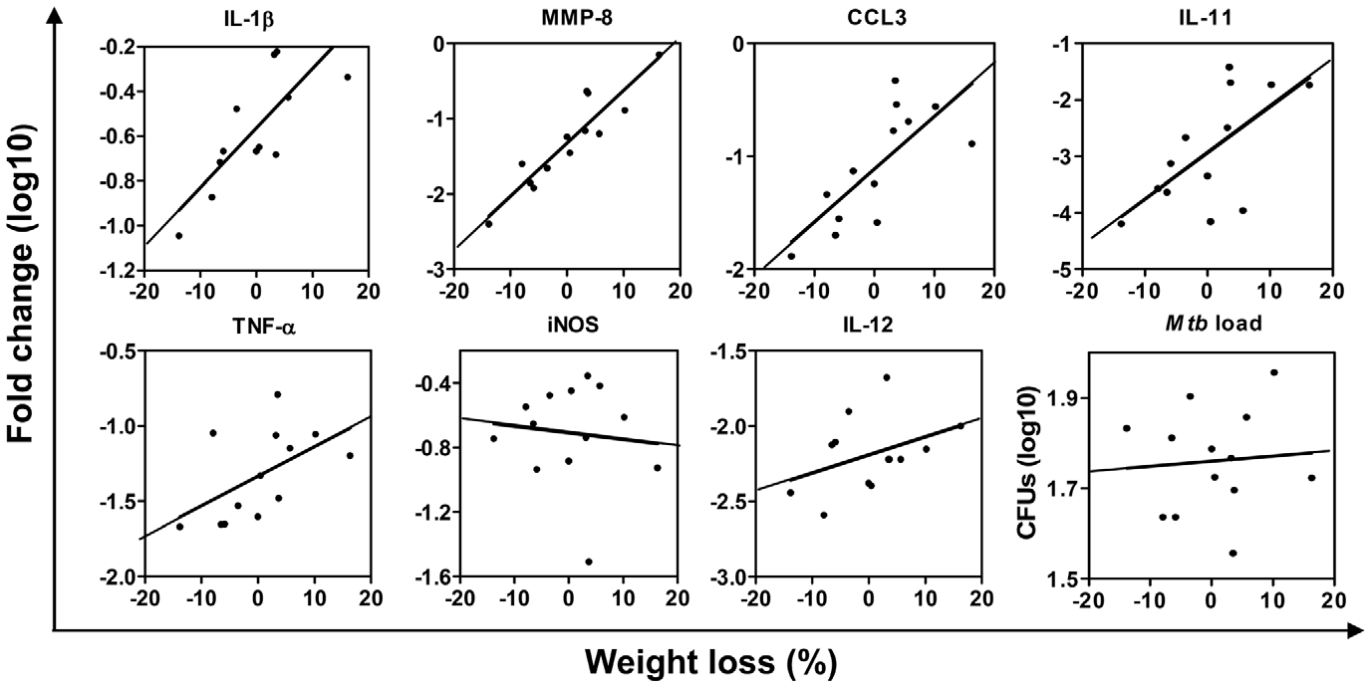
F2 mice exhibiting similar mycobacterial loads display different rates of TB progression that positively correlate with the lung inflammatory response. F2 mice challenged with *Mtb* were divided into several groups, each having similar *Mtb* loads. Correlations between wasting, mycobacterial load, and cytokine/chemokine expression in mice with lung mycobacterial burdens ranging from 3.3×10^7^ to 1×10^8^ CFUs/lobe are shown (for Spearman correlation coefficients and p-values see Table 3).

There is no doubt that the host inflammatory reaction is driven by pathogen-derived signals [21], [22], and that the intensity of the inflammatory response depends on the quantity of pathogen (i.e., *Mtb* load). We hypothesized that besides that, the inflammation intensity may depend on the reactivity of the host to the same pathogen load. To address this hypothesis, we re-analyzed our results obtained in F2 mice, by taking into account *Mtb* colonization data from each individual F2 mouse. The mice were divided into several groups, each group containing mice with similar *Mtb* loads (less than 3.3-fold differences), and the relative cytokine and chemokine expression was analyzed within each group. We found that despite similar *Mtb* loads, mice from each group displayed significant variability in the degree of wasting, and in the levels of inflammatory cytokine and chemokine expression (see Figure 4, for examples). In groups that included relatively high numbers of mice (n = 10 and more), a direct correlation between wasting and the expression of IL-1β and IL-11 was detected (Table 3). Correlations between wasting and lung expression of TNF-α, CCL3, and CXCL2 were different (i.e., positive, negative, or insignificant) depending on the selected group of mice, findings which likely mirrored the dual role for these cytokines in TB protection/TB pathology. In none of the analyzed groups was wasting correlated with *Mtb* load or the expression of iNOS.

**Table 3:** Correlation between wasting, mycobacterial load, and lung expression of cytokines and chemokines in F2 mice grouped by mycobacterial load.

Group of mice (#)		1	2	3	4	5	6
CFUs in group (×10^6^)		<3.3	3.3–10	10–33	33–100	100–333	>333
n		9	23	9	13	10	11
Mycobacterial load	ρ	0.18	−0.02	0.00	0.07	0.18	0.17
	p-value	6.0×10^−1^	9.0×10^−1^	1.0	8.3×10^−1^	6.0×10^−1^	7.8×10^−1^
IL-11	ρ	0.27	**0.50**	0.14	**0.66**	0.51	−0.54
	p-value	4.6×10^−1^	**1.4×10** ^−**2**^ *	7.4×10^−1^	**1.5×10** ^−**2**^ *	1.3×10^−1^	1.7×10^−1^
IL-1β	ρ	0.7	0.10	−0.08	**0.81**	**0.67**	**0.79**
	p-value	8.8×10^−1^	6.5×10^−2^	8.4×10^−1^	**8.0×10** ^−**4**^	**3.0×10^−3^**	3.0×10^−2^*
CCL3	ρ	−0.41	**−0.53**	0.05	**0.79**	**0.76**	**0.79**
	p-value	2.6×10^−1^	**1.2×10** ^−**2**^	9.0×10^−1^	**1.2×10** ^−**3**^	1.0×10^−2^*	3.0×10^−2^*
CXCL2	ρ	−0.47	**−0.48**	−0.35	**0.83**	**0.83**	0.67
	p-value	2.1×10^−1^	**2.0×10** ^−**2**^ *	3.5×10^−1^	**3.0×10** ^−3^	**3.0×10** ^−3^	8.0×10^−2^
TNF-α	ρ	−0.29	**−0.52**	−0.37	0.47	0.61	0.67
	p-value	4.4×10^−1^	**1.0×10** ^−**2**^ *	3.1×10^−1^	6.4×10^−2^	6.0×10^−2^	8.0×10^−2^
iNOS	ρ	−0.02	−0.37	−0.08	0.04	0.52	0.59
	p-value	9.8×10^−1^	8.0×10^−2^	8.4×10^−1^	8.8×10^−1^	1.6×10^−1^	1.3×10^−1^

Based on mycobacterial load, all F2 mice (the same as in Table 2) were divided into several groups so that within each group, CFU counts differed by no more than 3.3 fold. Correlations between wasting and CFU counts or wasting and lung expression of the indicated factors were determined separately for each group of mice. Note that the absence of significant correlation was observed mainly in groups with low numbers of mice (less than 10). ρ, Spearman coefficient; p-value, significant value of the test. Significant correlations (p<0.008) are shown in bold. Correlations with p-value>0.008 but less than 0.05 are marked with an asterisk.

These results showed that genetically different mice mounted inflammatory response of different intensity, even when harboring the same amount of mycobacteria in their lungs, and demonstrated that the intensity of this response correlated with TB progression. Thus, lung tissue inflammation can contribute to disease progression independently of *Mtb* load.

### Factors associated with TB progression are expressed by lung phagocytic cells

**Figure 5:**
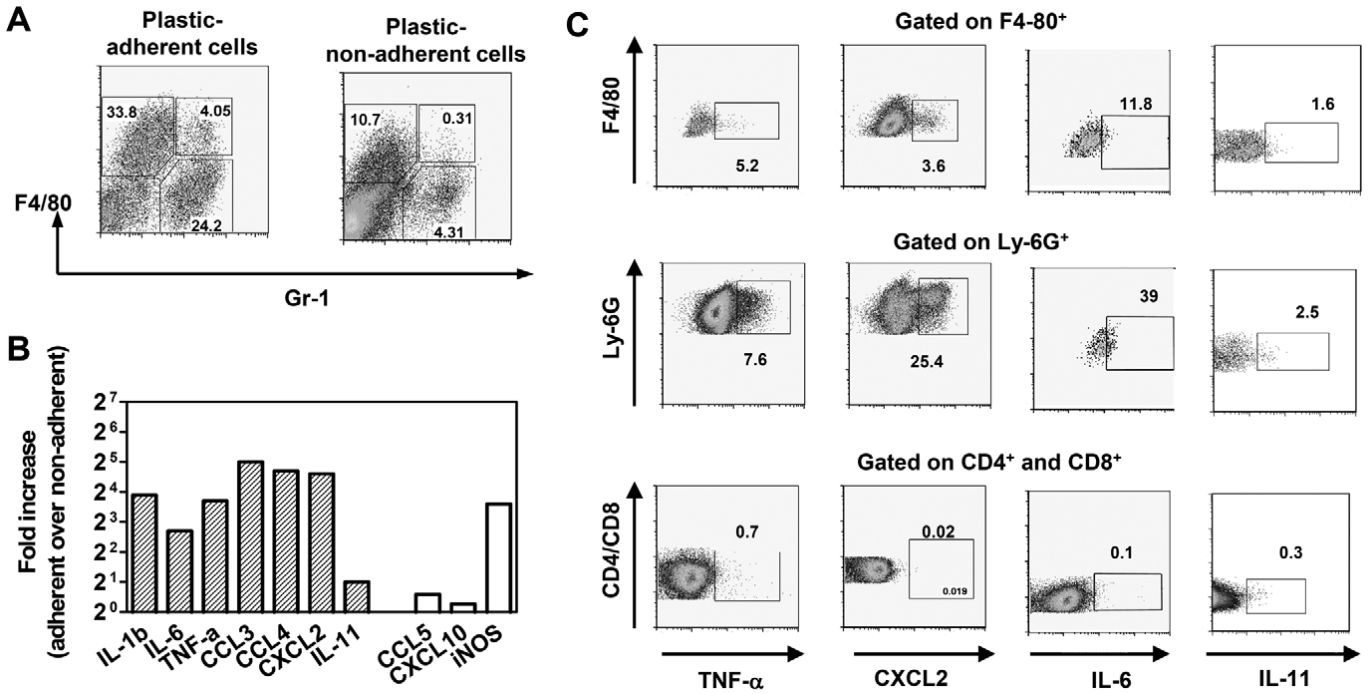
Pro-inflammatory factors associated with TB progression are expressed by lung phagocytic cells. F2 mice were challenged with *Mtb*. On day 24 post-infection, suspensions of lung cells isolated from moderately wasting mice were separated into plastic-adherent and non-adherent populations, and gene expression was analyzed by real-time PCR. A, Flow cytometry analysis of plastic-adherent and plastic-non-adherent populations. Cells were stained with mAbs specific to F4-80 and Gr-1 (clone RB6-8C5) antigens. Note enrichment for F4/80^+^ and Gr-1^+^ cells in plastic-adherent over non-adherent population (60% over 14%). B, Gene expression in plastic-adherent *versus* plastic-nonadherent populations (fold change in expression). Closed bars: pro-inflammatory factors associated with TB progression; open bars: factors that did not correlate with TB progression (Figure 3, Table 2 and data not shown). C, Production of TNF-α, CXCL2, IL-6, and IL-11 by different populations of lung cells (intracellular staining, two independent experiments). Cells were stained with mAbs specific to CD4, CD8, F4-80, and Ly-6G (clone 1A8) antigens. Gates are placed based on the fluorescence-minus-one control for each of analyzed subset.

To identify cells responsible for the expression of pro-inflammatory factors in the infected lungs, we used two experimental approaches. First, we separated lung cells derived from *Mtb*-infected F2 mice into plastic-adherent and plastic-non-adherent populations, and compared gene expression in these populations of cells. The cells were obtained from the lungs of F2 mice, 24 days following the challenge with *Mtb*. Flow cytometry analysis showed that plastic adherent population was enriched for F4-80^+^Gr-1^−^ (macrophages) and F4-80^−^Gr-1^+^ (presumably, granulocytes) cells that together formed more than 50% of the adherent cells (Figure 5A). In the plastic-non-adherent population, the proportion of phagocytes was significantly reduced (less than 15%, Figure 5A). In the adherent population, the expression of IL-1β, IL-6, TNF-α, CCL3, CCL4, CXCL2, as well as that of iNOS, was 8 to 30-fold higher relative to the non-adherent cells (Figure 5B), indicating lung phagocytes as the likely source of the analyzed factors.

Second, we assessed the production of several pro-inflammatory factors by different populations of lung cells, using intracellular cytokine staining. Lung cells were isolated from *Mtb*-infected F2 mice and stained with Abs specific to F4-80, Ly-6G, CD4/CD8 receptors and TNF-α, IL-6, CXCL2 or IL-11 (Figure 5C). TNF-α was found within F4-80^+^, Ly-6G^+^ cells and T-lymphocytes. CXCL2 and IL-6 were detected in both F4-80^+^ and Ly-6G^+^ cells, but not in T-lymphocytes. We noted that a particularly high proportion of Ly-6G^+^ cells (>20%) produced CXCL2. Given that this chemokine possesses neutrophil-attracting activity, these results suggested a positive feedback regulation of neutrophilic inflammation during TB. IL-11 was detected in a small percent of F4-80^+^ and Ly-6G^+^ cells, which was consistent with a low lung mRNA expression of this cytokine (see Figure 3).

Altogether, our results identified lung phagocytes as the major immune cells producing cytokines and chemokines associated with TB progression.

### Progressing TB is characterized by the accumulation of Ly-6G^dim^ cells in infected lungs

Given a role which F4-80^+^ and Gr-1^+^ cells played in the inflammatory response, we next examined whether these cells accumulated in different quantities in the lungs of mice with different rates of TB progression. Suspensions of lung cells were obtained from F2 mice, challenged 24 days prior the experiments, and analyzed by flow cytometry.

**Figure 6:**
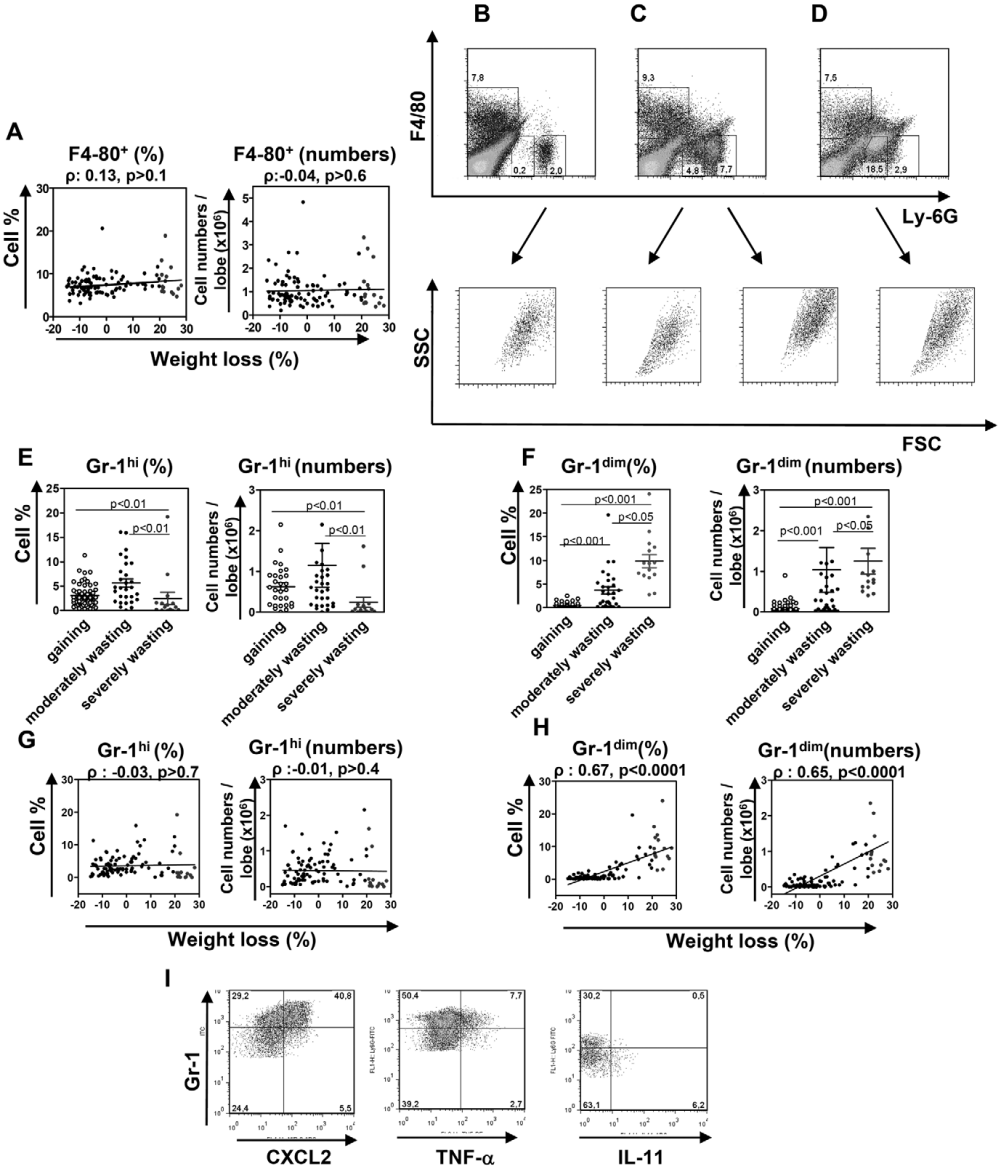
The accumulation of Gr-1^dim^ cells in the infected lungs is a characteristic feature of progressive TB. F2 mice were challenged with *Mtb* as described in Figure 1. On day 24, lung cell suspensions were obtained and stained with mAbs specific to F4-80 and Gr-1 (clone RB8-C6) or Ly-6G (clone 1A8) antigens (A–H). A, correlation between wasting, percentages and numbers of F4-80^+^Gr-1^−^ cells in the lungs. B–D, typical examples of flow cytometry analysis of cells derived from the lungs of gaining (B) or wasting (C, D) mice. Shown are results obtained with PE-anti-F4-80 and FITC-anti-Ly-6G Abs. E, F, The content of Gr-1^hi^ (E) and Gr-1^dim^ (F) cells in the lungs of gaining, moderately wasting and severely wasting mice. G, H, correlation between wasting, percentages and numbers of Gr-1^hi^ (G) and Gr-1^dim^ (H) cells in the lungs. Note that Gr-1^dim^ cells are negligible in gaining mice and that all mice with increased percentages or numbers of Gr-1^dim^ cells are wasting. I, Intracellular cytokines in Gr-1^hi^ and Gr-1^dim^ cells. Note that CXCL2 and TNF-α are produced mainly by Gr-1^hi^ cells, while IL-11 – by Gr-1^dim^ cells.

The percentages and the numbers of F4-80^+^Gr-1^−^ cells (macrophages) varied slightly between the mice, and did not correlate significantly with TB progression (Figure 6A). When analyzing the expression of Gr-1 marker, we noticed that Gr-1-positive cells were not uniform, but included cells with both high and low expression (Figure 6B–D). On Gr-1/Ly-6G *vs* F4-80 and FSC *vs* SSC dot plots, Gr-1^hi^ cells exhibited a distribution characteristic of neutrophils, and were Gr-1^hi^F4-80^−^ (Figure 6B); a population of Gr-1^dim^ cells was more diffuse and expressed Gr-1^dim^F4-80^−/dim^ phenotype (Figure 6 C,D). Gr-1^hi^ cells were present in the lungs of gaining and moderately wasting mice, but were almost absent from the lungs of severely wasting mice (Figure 6 D,E). In contrast, Gr-1^dim^ cells were negligible in gaining mice, but were readily identified in the lungs of wasting mice (Figure 6 F). In severely wasting mice, these cells became especially abundant (Figure 6 F) and co-expressed low levels of F4-80, i.e., were Gr-1^dim^F4-80^dim^ (Figure 6D). These results were first obtained using mAbs specific to Gr-1 antigen that bind both Ly-6G and Ly-6C molecules (clone RB6-8C5), but were also reproduced when mAbs specific to Ly-6G molecules only (clone 1A8) were used. The Gr-1^hi^/Ly-6G^hi^ cells likely represented neutrophils [23]. The nature of Gr-1^dim^/Ly-6G^dim^ cells was less clear, but it was very unlikely that they could be classified as “normal” mature neutrophils. That these cells represented dying neutrophils was also unlikely, as their FSC-SSC distribution and Annexin V binding were similar to those of Gr-1^hi^ cells (Figure 6 B–D and data not shown). Rather, Gr-1^dim^ cells represented early neutrophil precursors, or modified neutrophils that were generated in highly inflammatory conditions and replaced typical neutrophils (see discussion for the details).

Correlation analysis performed separately for Gr-1^hi^ and Gr-1^dim^ cells showed that the accumulation of Gr-1^dim^, but not Gr-1^hi^, cells correlated with TB progression (Figure 6 G, H). The fact that Gr-1^dim^ cells were negligible in all gaining mice (Figure 6F) indicated that the appearance and the accumulation of these cells was a characteristic cellular feature of progressing TB.

Analysis of the roles for Gr-1^hi^ and Gr-1^dim^ cells in the production of certain pro-inflammatory cytokines showed that CXCL2 and TNF-α were produced mainly by Gr-1^hi^ cells (neutrophils). In contrast, IL-11 was found in some Gr-1^dim^ cells, but was almost absent from Gr-1^hi^ cells (Fig. 6I).

These studies showed that high expression of pro-inflammatory factors in the lungs of mice with rapidly progressing TB could not be attributed to a higher number of macrophages and only in part (in some moderately wasting mice) could be attributed to the accumulation of neutrophils in the lungs. The accumulation of Gr-1^dim^ cells could account for the increased expression of some (e.g., IL-11), but not all factors associated with TB progression. Importantly, these cells were absent from the lungs of un-infected mice (data not shown), i.e. they could not be responsible for the initiation of the local inflammatory reaction.

### Genetically different phagocytes differ intrinsically by the expression of factors associated with TB progression

In the next set of experiments we analyzed whether phagocytes from TB-susceptible and resistant mice differed in their expression of inflammation-related factors at a per cell level.

**Figure 7:**
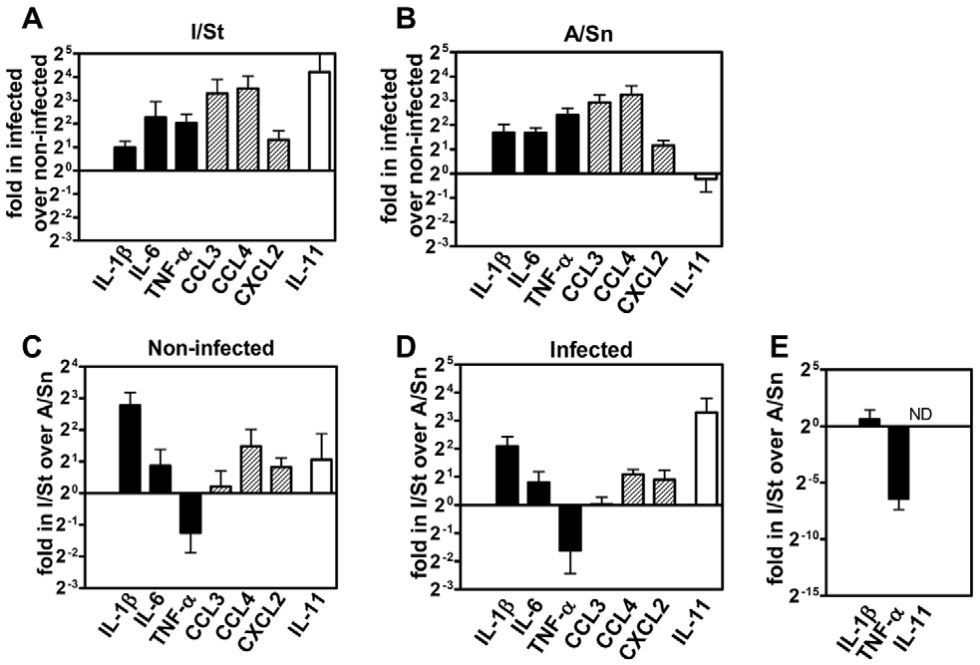
I/St and A/Sn macrophages and neutrophils differ in the expression of pro-inflammatory cytokines. A–D, Analysis of gene expression in macrophages. Macrophages were obtained from the peritoneal cavity of I/St and A/Sn mice, cultured with or without *Mtb*, and used for gene expression analysis. A, B, Gene expression in infected *versus* uninfected macrophages from I/St (A) and A/Sn (B) mice. C, D, Gene expression in I/St *versus* A/Sn macrophages, either uninfected (C) or infected (D). Data from one of three similar experiments are shown. E, Analysis of gene expression in neutrophils. Neutrophils were obtained from the peritoneal cavity of I/St and A/Sn mice and used for gene expression. Shown are gene expressions in I/St *versus* A/Sn neutrophils (results of three independent experiments). Bars show the relative expression of corresponding genes in infected *versus* un-infected phagocytes (A, B) or in I/St versus A/Sn phagocytes (C–E).

Macrophages were obtained from the peritoneal cavity of I/St and A/Sn mice, and were cultured *in vitro*, in the presence or absence of *Mtb*. In response to *Mtb* infection, both I/St and A/Sn macrophages up-regulated the expression of the pro-inflammatory genes IL-6, TNF-α, CCL3, CCL4, CXCL2 (Figure 7 A,B), which is consistent with other reports [24]. In both un-infected and infected cultures, I/St and A/Sn macrophages exhibited significant inter-strain differences in gene expression. The most striking differences were higher expression of IL-11 and IL-1β in “susceptible” I/St macrophages and a higher expression of TNF-α in A/Sn macrophages (Figure 7 C, D). Of note, in F2 mice, high expression of IL-11 and IL-1β and low expression of TNF-α (when the expression of IL-11 and IL-1β was taken into account) were the major correlates of TB progression (Files S2, S3, S4).

We next monitored the expression IL-1β, IL-11 and TNF-α in neutrophils (Figure 7E). The expression of IL-1β did not show stable inter-strain differences. The expression of IL-11 was very low in I/St neutrophils (Ct more than 38), and was undetectable in A/Sn neutrophils (no signal during 45 amplification cycles). The expression of TNF-α was stably lower in I/St as compared to A/Sn neutrophils, in four independent experiments.

Altogether, the data demonstrated that phagocytes, especially, macrophages, from I/St and A/Sn mice exhibited intrinsic differences in the expression of genes that were the major correlates of TB progression in F2 mice, suggesting that the peculiarities of phagocyte transcriptional response could account for the peculiarities of gene expression in the infected lungs and affect the outcome of *Mtb* infection.

## Discussion

Studies over the last several decades have demonstrated that a complete deficiency in factors mediating antibacterial response results in severe mycobacterial infections [4]–[6]. These studies supported the concept that TB disease develops as a result of ineffective antibacterial immune response. On the other hand, for many years TB had been considered as an immunopathological disease, in which pathology develops due to uncontrolled host inflammatory reactivity to the pathogen. Direct evidence for this concept had not been available, largely because the role that dysregulated inflammation plays in TB pathology is difficult to address. Indeed, the majority of factors mediating inflammation are prerequisite for the development of protection; therefore their targeting or neutralization results in disease exacerbation and masks potential pathological properties. In the current study, we took advantage of a mouse model of TB which we have utilized for several years. In this model, mice of I/St and A/Sn strains differ in their intensity of both antibacterial and inflammatory responses. The model has allowed us to address the relative roles for these responses in TB pathology, by analyzing their segregation and TB progression in (A/SnxI/St)F2 mice.

**Figure 8:**
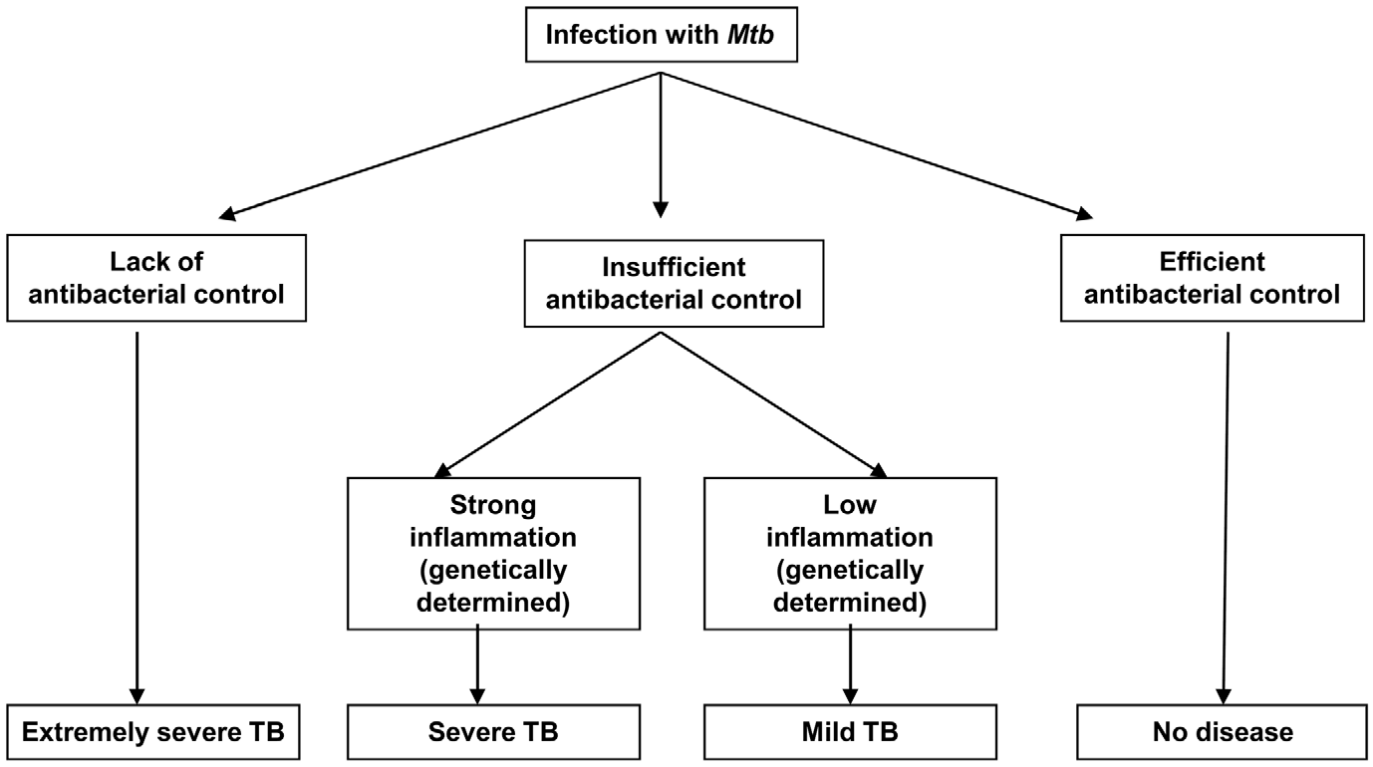
Suggested roles for antibacterial and inflammatory responses in the determination of TB outcome. In hosts with deficiency in antibacterial immune response, progressive *Mtb* growth induces extremely severe TB. In hosts able to eradicate *Mtb*, no disease is developed. In hosts who are able to restrict *Mtb* growth but fail to completely eradicate the infection, the outcome depends on quantitatively- and genetically-determined peculiarities of inflammatory response.

We find that in immunocompetent hosts TB progression is not a direct consequence of high *Mtb* loads, but rather, is a result of excessive inflammatory reaction developed in *Mtb* infected lungs. This is supported by the following observations. First, not all F2 mice with high mycobacterial burden in the lungs rapidly progressed to fatal infection. In most mice, the rate of TB progression did not depend on bacterial burden. Second, TB progression did not correlate with lung expression of factors involved in antibacterial response (iNOS, IFN-γ), but correlated with lung expression of factors involved in the development of inflammation (IL-1β, CXCL2, IL-11 et al.). Third, only one of the three QTLs involved in the control of TB severity in F2 mice, was implicated in the control of *Mtb* colonization in these mice. Our results correspond well to the study of Kramnik's group [19], which showed that differences in TB susceptibility between *sst-1* congenic mice did not depend on iNOS/NO production, but rather, were associated with the development of necrotic lung inflammation. Similarly, Bishai et al [25] demonstrated that immunopathology and lethality of TB did not depend on the capacity of mycobacteria to grow and survive in infected host. These reports, together with the results of our study, strongly indicate that host capacity to restrict *Mtb* growth is not the sole (and likely not the major) factor that determines TB outcome. Our view on the roles that antibacterial and inflammatory responses may play in the determination of TB outcome is presented on Figure 8.

Gene expression analysis performed in our study showed that factors expressed in the lungs of F2 mice segregated into two major groups: those not correlated with TB progression (iNOS, IL-12p40, IFN-γ, CCL5), and those associated with TB progression (IL-1β, IL-6, CCL3, CCL4, CXCL2, MMP-8, MMP-10, Figure 2 and data not shown). Analysis of macrophage transcriptional responses performed by other investigators revealed that, based on the up-stream regulatory pathways, cytokines and chemokines may be similarly segregated into two groups: “antibacterial” (iNOS, CCL5 et al) and “inflammatory” (IL-1β, CCL3, CCL4, CXCL2). Almost all factors that in our study were associated with TB progression (IL-1β, CCL3, CCL4, CXCL2) were considered as “inflammatory”, and were reported to be induced via TLRs. In contrast, factors not associated with TB progression in our model (e.g., iNOS, CCL5) were considered as antibacterial and were largely induced by TLR-independent IFNαβγ-dependent pathways [22], [24], [26]. Thus, there was a close association between the involvement of genes in TB progression (this study), their participation in host inflammatory response, and the regulation of their expression/production via TLR-dependent pathways [22], [24], [26]. This conclusion is further supported by recent observations in humans that demonstrated that S180L polymorphism in TIRAP gene, which leads to the attenuation of inflammation (TIRAP is implicated in the TLR2- and TLR4- mediated signaling pathways), is associated with a decreased risk of TB development [27].

In our study, cytokine expression was evaluated at mRNA level. Because the production of some cytokines, e.g., IL-1β, is regulated posttranslationally, their differential transcription could be relevant or not to the secretion of biologically active protein. We believe that in our model, higher expression of IL-1β mRNA was relevant to a higher production of IL-1β protein and did contribute to disease progression: when we analyzed the amount of IL-1β protein in the lungs of some F2 mice, we found that mice exhibiting higher expression of IL-1β mRNA had higher amounts of IL-1β protein, and the later correlated with the degree of wasting (r = 0.61, p<0.06; r = 0.82, p = 0.004, respectively, data not shown). These results are in line with the observations made in humans that showed an increased release of IL-1β, IL-6, and TNF-α in the BAL fluid of patients with active TB, as compared to healthy controls [28], and higher concentrations of IL-1β in BAL fluids of patients with large cavities related to patients with small or no cavity [29]. Our conclusion as to a possible role of inflammatory responses in TB progression does not contradict data on severe course of TB in mice deficient in IL-1β, IL-6 or IL-1β receptor [30]–[32]. As discussed above, pro-inflammatory factors likely play a dual role during TB, and may mediate protection when produced in low doses or induce pathology when secreted at high doses. Interestingly, in mice lacking the IL-1R, a severe course of TB was associated with high lung production of IL-1β, IL-6, and TNF-α [32], findings which corresponds well to the result of our study.

An important question is how the intensity of lung inflammatory response is controlled during TB. In phagocytes, inflammatory response is induced by pathogen-derived signals [21], [22], [24], i.e. pathogen load is one of the major factors that determine the intensity of inflammation. Our results indicate that other factors, in particular genetics, may be also implicated in the control of inflammatory response during TB. Indeed, F2 mice having similar mycobacterial loads in their lungs displayed different levels of inflammatory factor expression. The levels of inflammatory factors expression, rather than *Mtb* loads, correlated with TB progression in F2 mice (Tables 2, 3). Genetically different macrophages and neutrophils displayed different levels of expression of inflammation-related genes, and these differences were seen not only in *Mtb*-infected but also in *Mtb*-un-infected cells. Thus, variations between individual F2 mice in the intensity of lung inflammatory response cannot be explained in terms of the host ability to restrict mycobacterial growth, and may be due either to stochastic fluctuations, or to genetically controlled differences between the mice. Giving that macrophages from parental I/St and A/Sn strains displayed intrinsic differences in the expression of inflammatory genes, and that the same two cytokines, IL-1β and IL-11, were highly expressed in “susceptible” I/St macrophages, and in the lungs of F2 mice with severe TB, we believe that the second explanation is more likely, i.e. the intensity of inflammatory response mounted by infected F2 mice were controlled genetically. This assumption is consistent with the results of Keller et al [33] who showed that macrophages from TB-susceptible mice are characterized predominantly by the activation of genes involved in local inflammatory responses, and is further supported by the data from Poltorak and coauthors [34], who directly demonstrated a role for genetic factors in the control of macrophage inflammatory responses.

In our study, the best correlates of TB progression were high expressions of IL-1β and IL-11. An association between TB progression and the expression of IL-11 is a novel finding of this study. This finding extents previous observations on a higher expression of IL-11 mRNA in I/St macrophages [20] and raises a question on whether this cytokine is directly implicated on TB pathogenesis and if so, which mechanisms mediate IL-11 effect. The answer to these questions requires long-term experiments on IL-11 administration and/or its neutralization following *Mtb* infection, which were beyond of this study and are currently ongoing. Based on the results obtained in other experimental models, we suppose that several biological activities of IL-11 are relevant to TB control. In particular, IL-11 was shown to shift T-cell response towards a Th2 type [35], [36], modify hemopoiesis [35], [37], [38], and directly affect inflammation [35], [39]–[41]. Of note, an ability of IL-11 to up-regulate the expression of IL-1β mRNA and IL-1β-dependent up-regulation of IL-11 were reported [42], [43], indicating on the ability of IL-1β and IL-11, the two major correlates of TB progression in our study, to positively regulate each other.

Analysis of the expression of TNF-α revealed steady intrinsic differences between macrophages and granulocytes derived from I/St and A/Sn mice. These results strongly suggest that the level of TNF-α expression is under genetic control. In I/St macrophages, a relatively low expression of TNF-α paralleled with a relatively high expression of IL-11, which is in line with a known capacity of IL-11 to down-regulate TNF-α [39]. In F2 mice, the expression of TNF-α correlated directly with TB progression when simple correlation analysis was performed in all F2 mice or in F2 mice having high *Mtb* loads. However the expression of TNF-α correlated negatively with TB progression when the expression of other factors, especially, IL-1β and IL-11, was taken into account or when simple correlation analysis was performed in mice having low *Mtb* loads. These results show that high expression of TNF-α may be protective or contribute to pathology depending on pathogen burden and/or local inflammatory background, which is in line with multiple roles for this cytokine in TB pathogenesis, and its capacity to both promote and down-regulate the inflammation [44].

Earlier we and other investigators demonstrated that in mice, severe TB was accompanied by a progressive accumulation of neutrophils in the lung tissue [18], [45], [46]. In these studies, neutrophils were identified as cells that bind Gr-1-specific (RB6-8C5) Abs. In the current study, we demonstrate that the population of Gr-1^+^ cells infiltrating *Mtb*-infected lungs, is not homogeneous, and consists of at least two different subsets, Gr-1^hi^ and Gr-1^dim^. We show that TB progression is tightly associated with the appearance and progressive accumulation of Gr-1^dim^/Ly-6G^dim^, but not Gr-1^hi^/Ly-6G^hi^, cells in the lungs, which is a novel finding of this study. While there are no doubts that Gr-1^hi^/Ly-6G^hi^ cells represent neutrophils, the nature of Gr-1^dim^/Ly-6G^dim^ cells is not completely clear. Gr-1-specific Abs produced by clone RB6-8C5, recognize an epitope shared by Ly-6G and Ly-6C molecules. Ly-6G-specific Abs produced by clone 1A8 recognize specifically Ly-6G molecules. Granulocytes are characterized by the expression of Ly-6G protein and are known to express the Gr-1^hi^/Ly-6G^hi^ phenotype [23]. Monocytes do not express Ly-6G, but express Ly-6C protein, and therefore are Gr-1^low^/Ly-6G^−^ [47]. Gr-1^dim^/Ly-6G^dim^ cells identified in our study differed from typical monocytes as they could be stained with Ly-6G Abs. On the other hand, these cells also differed from typical granulocytes as their expression of Gr-1 and Ly-6G was relatively low. In addition, these cells co-expressed low levels of F4-80 (Figure 6D) and had un-segmented nuclei (Lyadova, Barteneva, unpublished observations). We suppose that Gr-1^dim^/Ly-6G^dim^ cells, identified in our study, represent immature granulocytes or their myeloid precursors that generate and migrate to peripheral tissues in highly inflammatory conditions. It is also possible that Gr-1^dim^/Ly-6G^dim^ cells represent cells developed from mature neutrophils as a result of their transformation at the site of inflammation. The later is in line with recent observations made by Sasmono et al [48] who suggested that at inflammatory sites, neutrophils may differentiate into F4-80 expressing macrophages and that this transdifferentiation is accompanied by down-regulation of Ly-6G expression. Although the exact nature of Gr-1^dim^/Ly-6G^dim^ cells remains to be established, there is no doubt that in our model, the accumulation of these cells in the lungs was the major cellular characteristic of TB progression.

In our study, the numbers of neutrophils (Gr-1^hi^ cells) in the lungs of mice with rapidly progressing TB were not higher compared to mice with slowly progressing disease (Figure 6). These results seem to contradict numerous observations (including our own) demonstrating that severe TB is accompanied by a disproportional influx of neutrophils to *Mtb*-infected lungs [18], [45], [46]. The contradiction is likely due to the fact that in other studies the levels of Gr-1 expression were not taken into account and all Gr-1-expressing cells were considered as typical neutrophils.

An association between the accumulation of phagocytic cells, high expression of pro-inflammatory cytokines and chemokines in the lungs, and TB severity, observed in our study, is in line with the results obtained in other mouse models of TB [46]. Our results suggest that although phagocytic cells can contribute to the propagation of inflammation, their progressive accumulation in the lungs is rather a result than an initial cause of severe inflammation. Indeed, in our study, the numbers of macrophages and neutrophils (the major immune cells producing pro-inflammatory cytokines) in the lungs of mice with progressive TB were not higher compared to mice with slowly progressing disease (Fig. 6). Gr-1^dim^/Ly-6G^dim^ cells were abundant in the lungs of mice with severe TB, but these cells were poor producers of major pro-inflammatory factors (besides IL-11) and were absent form the lungs of un-infected mice, i.e. could not serve as an initial cause of inflammation. Finally, neutrophils and, especially, macrophages from I/St and A/Sn mice exhibited intrinsic differences in the expression of pro-inflammatory factors at a per cell level (Fig. 7 C, D). Thus, we suppose that excessive expression of inflammation-related factors in the lungs of mice with severe TB depended on the peculiarities of phagocytes transcriptional response to the infection at a per cell level, rather than was a result of their massive local accumulation.

*In conclusion*, this study provides evidence that severe rapidly progressing TB may result not only from insufficient effector functions, but also be a consequence of excessive inflammatory responses in infected hosts, supporting the idea that excessive inflammation may be more damaging for the host than the activity of the pathogen that elicited it.

Another important conclusion of this study is that in a “normal” immunocompetent population of genetically heterogeneous hosts, the outcome of *Mtb* infection is determined by “subtle” variations in host immune reactivity (e.g., by the quantitative differences in the level of inflammatory gene expression), rather than by “on-off” differences (e.g., expression or a complete lack of expression of a particular factor). In our study, variations in lung expression of cytokines IL-1β and IL-11 were the major factor associated with TB progression in mice. Whether IL-1β and, especially, IL-11, a cytokine with previously unidentified role during TB, account for severe course of TB in humans, is an important question. Finally, our study, for the first time, identifies an unusual population of Gr-1^dim^/Ly-6G^dim^ cells, which accumulation in the infected lungs marks TB progression.

## Materials and Methods

### Animals

(A/Sn×I/St)F2, A/JSnYCit (A/Sn), and I/StYCit (I/St) mice were bred in the Animal Care Facility at the Central Tuberculosis Research Institute (Moscow, Russia) in accordance with Russian Ministry of Health Guideline no. 755 and the US National Institutes of Health Office of Laboratory Animal Welfare (OLAW) Assurance #A5502-01. Water and food were provided ad libitum. All experimental procedures were approved by the CIT IACUC.

### Study design

Mice were infected intratracheally with 10^3^ CFUs/mouse of mid-log-phase *Mtb* strain H37Rv Pasteur as described earlier [16]. F2 mice were weighted before the infection, and then every 7 days.

Experiments were performed on day 24 post-infection. Lungs were perfused with 0,02% EDTA-PBS to wash blood vessels [11] and used for: 1) determination of mycobacterial load, 2) histological analysis, 3) flow cytometry analysis, and 4) RNA isolation. Mycobacterial loads were determined by plating homogenates of the upper right lobe of the lungs on Dubos agar [11]. Lung tissue pathology was evaluated by preparing serial 6- to 8-µm sections of the left lobe of the lung. The sections were stained with haematoxylin-eosin and analyzed using an Axioskop 40 microscope (magnification 2.5× and 40×) equipped with AxioCam MRc 5 camera (Carl Zeiss, Germany). The area of infiltrated lung tissue was determined using AxioVs 40 software. The percentage of lung tissue affected by TB was calculated as: infiltrated lung tissue area (µm^2^)/(total lung tissue area (µm^2^)×100%. For flow cytometry analysis, the bottom-right lobes of the lung were excised, digested, and analyzed using FACS Calibur with CellQuest (BD Bioscience, San Jose, CA) and Flow Jo (TreeStar, Inc. San Carlos, CA) softwares. Lung cell viability was determined based on cell distribution on FSC-SSC dot plots and by tripan blue exclusion, which gave similar results. mRNA was isolated from the middle right lobe of each lung using SV Total RNA Isolation System (Promega, CA). For the determination of weight loss, *Mtb* load in the lungs, lung pathology, and lung cell viability mice of both sexes were used. Gene expression analysis was performed in females to exclude any influence of gender on the results.

### Isolation of plastic-adherent, plastic-non adherent lung cells and peritoneal macrophages

Lung cell suspensions were obtained from moderately wasting or weight gaining F2 mice on day 24 post-infection. Cells were pooled within each group. Plastic-adherent and non-adherent populations were separated as described earlier for macrophages [17]. RNA was isolated from 3×10^6^ of non-adherent or adherent cells using SV Total RNA Isolation System (Promega). Aliquots of non-adherent and adherent cells were analyzed by flow cytometry to confirm their phenotype.

### Gene expression in macrophages and neutrophils

I/St and A/Sn mice were injected intraperitoneally with 3% peptone. To obtain macrophages, peritoneal exudate cells were eluted five days later and plastic-adherent population was isolated [17]. The cells (4–5×10^6^ cells/well in 6 ml) were placed in 6-well tissue culture plates at 37°C in 5% CO2; one hour later mycobacteria were added at a multiplicity of infection of 2∶1. After incubation for 18h, the non-adherent cells were eliminated by vigorous washing and macrophage mRNA was isolated. To obtain neutrophils, peritoneal exudate cells were eluted 18 h after the injection of peptone. Neutrophils were purified using two-step Percoll gradient (1.073 and 1.100 g/ml) and immediately used for mRNA isolation.

### Quantitative RT-PCR

RNA was reverse transcribed to generate cDNA using oligo (dT) primers, dNTP mix, RNaseOUT, and SuperScript II reverse transcriptase (Invitrogen, Carlsbad, CA), following manufacturer's protocol. The cDNA was used as the template for quantitative PCR using an ABI Prism 7000 Sequence Detection System (Applied Biosystems, Foster City, CA, USA). Gene expression in the lungs was analyzed using glyceraldehyde 3-phosphate dehydrogenase (GAPDH) as a housekeeping gene, as it was uniformly expressed in the lungs of mice with different TB severity. In *in vitro* cultured cells, β-Actin and hypoxanthine ribosyltransferase (HPRT), but not GAPDH, revealed stable expression and were used as reference genes. Gene expression assays for GAPDH, β-Actin, IL-1β, TNF-α, CCL3, CCL4, CXCL2, iNOS, were purchased from Applied Biosystems. Primers and probes for IL-11, IL-6, MMP-8, MMP10 were designed by Pamela Scott Adams (Trudeau Institute Inc., Saranac Lake, NY), and for HPRT - by Dr. Gregory Dolganov (Stanford University, Stanford, CA). The sequences of these primers and probes are as follows. IL-11: forward TACTCCGCCGTTTACAGCTC, reverse GGGGATCACAGGTTGGTCT, probe ATGTCTCGCCTGGCCTTGCC; IL-6: forward GTTCTCTGGGAAATCGTGGA, reverse AAGTGCATCATCGTTGTTCATACA, probe TGAGAAAAGAGTTGTGCAATGGCAATTCTG; Mmp-8: forward AGGGAACCCAGCACCTATTC, reverse CAGTAGGTTGGATGGGGTTG, probe CAATGGCATTCAGACAATCTATGGACC; Mmp10: forward, GTGATCCTGCTTTGTCCTTTG, reverse TGAAATTCAGGCTCGGGATT, probe CTTTAAAGACAGGTACTTCTGGCGCAGATCCC; HPRT: forward CTTCCTCCTCAGACCGCTTTT, reverse AACCTGGTTCATCATCGCTAATC, probe AGCCGACCGGTCCCGTCATG.

### Flow cytometry and intracellular cytokine staining

Lung cell suspensions obtained from F2 mice were stained with PE-anti-F4-80 (Caltag) and FITC-anti-Gr-1 (clone RB6-8C5) or FITC-anti-Ly-6G (clone 1A8) mAbs (BD Bioscience). For detection of intracellular cytokines, lung cells obtained from F2 mice were cultured in the presence of *Mtb* sonicate and GoldgiPlug (BD Bioscience), and stained as described earlier [49] using the following mAbs: FITC-anti-Ly-6G (clone 1A8), FITC- or PE-anti-F4/80, Per-CP-anti-CD4, Per-CP-anti-CD8, PE-anti-TNF-α APC-anti-IFN-γ (BD Biosciences), biotinilated-anti-CXCL2, biotinilated-anti-IL-6 or biotinilated-anti-IL-11 Abs (R&D Systems Inc., Minneapolis, MN) plus streptavidin-FITC or streptavidin-APC. Isotype-matched antibodies were used as controls.

### DNA preparation and QTL analysis

Tail DNA was isolated from (A/Sn×I/St)F2 mice using a Wizard Genomic DNA Purification kit (Promega) and used for the determination of the simple sequence length polymorphism (SSLPs) D3Mit299, D9Mit89, and D17Mit175 (MapPairs, Research Genetics, Massachusets) as described previously [8], [14]. QTL analysis was performed using mycobacterial load and percent of IFN-γ-producing cells in the lungs as quantitative traits (QTX MapManager, Software for genetic mapping of mendelian markers and quantitative trait loci, program for Windows).

### Statistical analysis

Correlations between quantitative variables were performed using Spearman analysis (GraphPad Software, Inc., San Diego, CA and program R, http://www.r-project.org). Selection of the best model predicting disease progression was done by multiple regression analysis, using F-test for nested models, and Akaike Information Criterion [50]–[52]. Before the analysis, the gene expression data were log-transformed with a detection limit of 1×10^−5^. The positive ρ (rho) indicated the direct, and the negative ρ (rho) – inverse correlation. For the multiple correlation analyses, p-values of 0.003 (12 variables) or 0.006 (8 variables) were considered significant to account for multiple testing.

## Supporting Information

File S1 Removing severely wasting mice from the analysis.
(0.02 MB DOC)

File S2 Multiple regression analysis and F-tests.
(0.02 MB DOC)

File S3 Selecting a minimal model. Akaike and Bayesian Information Criterion.
(0.02 MB DOC)

File S4 Bootstrapping to account for variability.
(0.02 MB DOC)

Figure S1 Correlation between disease progression (as determined by the weight loss at day 24 post-infection) and *Mtb* load for female mice. Dashes blue line, the prediction of the linear regression between weight loss and *Mtb* load when all mice are included in the analysis; solid black line, the same when severely wasting mice (shown by blue crosses) are excluded from the analysis.
(4.03 MB TIF)

Table S1 Results of the linear regression analysis of the weight loss and all factors in gaining and moderately wasting mice.
(0.04 MB DOC)

## References

[1] Raviglione MC (2003). The TB epidemic from 1992 to 2002.. Tuberculosis (Edinb).

[2] Bellamy R, Hill AVS (1998). Host genetic susceptibility to human tuberculosis.. Novartis Foundation Symposium.

[3] Dietrich WF (2001). Using mouse genetics to understand infectious disease pathogenesis.. Genome Res.

[4] Flynn JL, Ernst JD (2000). Immune responses in tuberculosis.. Curr Opin Immunol.

[5] Collins HL, Kaufmann SH (2001). The many faces of host responses to tuberculosis.. Immunology.

[6] North RJ, Jung Y-J (2004). Immunity to tuberculosis.. Ann Rev Immunol.

[7] Winslow GM, Cooper A, Reiley W, Woodland DL (2008). Early T cell responses in tuberculosis immunity.. Immunol Rev.

[8] Lavebratt C, Apt AS, Nikonenko BV, Schalling M, Schurr E (1999). Severity of tuberculosis in mice is linked to distal chromosome 3 and proximal chromosome 9.. J Infect Dis.

[9] Kramnik I, Dietrich WF, Demant P, Bloom BR (2000). Genetic control of resistance to experimental infection with virulent *Mycobacterium tuberculosis*.. Proc Natl Acad Sci U S A.

[10] Nikonenko BV, Averbakh MM, Lavebratt C, Schurr E, Apt AS (2000). Comparative analysis of mycobacterial infections in susceptible I/St and resistant A/Sn inbred mice.. Tuber Lung Dis.

[11] Lyadova IV, Eruslanov EB, Yeremeev VV, Majorov KB, Nikonenko BV (2000). Comparative analysis of T lymphocytes recovered from the lungs of mice genetically susceptible, resistant and hyperresistant to *Mycobacterium tuberculosis*-triggered disease.. J Immunol.

[12] Mitsos LM, Cardon LR, Ryan L, LaCourse R, North RJ (2003). Susceptibility to tuberculosis: a locus on mouse chromosome 19 (Trl-4) regulates *Mycobacterium tuberculosis* replication in the lungs.. Proc Natl Acad Sci U S A.

[13] Cardona PJ, Gordillo S, Diaz J, Tapia G, Amat I (2003). Widespread bronchogenic dissemination makes DBA/2 mice more susceptible than C57BL/6 mice to experimental aerosol infection with *Mycobacterium tuberculosis*.. Infect Immun.

[14] Sanchez F, Radaeva TV, Nikonenko BV, Persson AS, Sengul S (2003). Multigenic control of disease severity after virulent *Mycobacterium tuberculosis* infection in mice.. Infect Immun.

[15] Pan H, Yan BS, Rojas M, Shebzukhov YV, Zhou H (2005). Ipr1 gene mediates innate immunity to tuberculosis.. Nature.

[16] Eruslanov EB, Majorov KB, Orlova MO, Mishenko VV, Kondratieva TK (2004). Lung cell responses to *M. tuberculosis* in genetically susceptible and resistant mice following intratracheal challenge.. Clin Exp Immunol.

[17] Majorov KB, Lyadova IV, Kondratieva TK, Eruslanov EB, Rubakova EI (2003). Different innate ability of I/St and A/Sn mice to combat virulent *Mycobacterium tuberculosis*: phenotypes expressed in lung and extrapulmonary macrophages.. Infect Immun.

[18] Eruslanov EB, Lyadova IV, Kondratieva TK, Majorov KB, Scheglov IV (2005). Neutrophil responses to *Mycobacterium tuberculosis* infection in genetically susceptible and resistant mice.. Infect Immun.

[19] Yan B-S, Pichugin AV, Jobe O, Helming L, Eruslanov EB (2007). Progression of Pulmonary Tuberculosis and Efficiency of Bacillus Calmette-Guérin Vaccination Are Genetically Controlled via a Common sst1-Mediated Mechanism of Innate Immunity.. J Immunol.

[20] Orlova MO, Majorov KB, Lyadova IV, Eruslanov EB, M'lan CE (2006). Constitutive differences in gene expression profiles parallel genetic patterns of susceptibility to tuberculosis in mice.. Infect Immun.

[21] Taylor PR, Martinez-Pomares L, Stacey M, Lin HH, Brown GD (2005). Macrophage receptors and immune recognition.. Annu Rev Immunol.

[22] Ehrt S, Schnappinger D, Bekiranov S, Drenkow J, Shi S (2001). Reprogramming of the macrophage transcriptome in response to interferon-gamma and *Mycobacterium tuberculosis*: signaling roles of nitric oxide synthase-2 and phagocyte oxidase.. J Exp Med.

[23] Hestdal K, Ruscetti FW, Ihle JN, Jacobsen SE, Dubois CM (1991). Characterization and regulation of RB6-8C5 antigen expression on murine bone marrow cells.. J Immunol.

[24] Shi S, Blumenthal A, Hickey CM, Gandotra S, Levy D (2005). Expression of many immunologically important genes in Mycobacterium tuberculosis-infected macrophages is independent of both TLR2 and TLR4 but dependent on IFN-alphabeta receptor and STAT1.. J Immunol.

[25] Kaushal D, Schroeder BG, Tyagi S, Yoshimatsu T, Scott C (2002). Reduced immunopathology and mortality despite tissue persistence in a Mycobacterium tuberculosis mutant lacking alternative sigma factor, SigH.. Proc Natl Acad Sci U S A.

[26] Heldwein KA, Liang MD, Andresen TK, Thomas KE, Marty AM (2003). TLR2 and TLR4 serve distinct roles in the host immune response agaoinst Mycobacterium bovis BCG.. J leukoc Biol.

[27] Castiblanco J, Varela DC, Castaño-Rodríguez N, Rojas-Villarraga A, Hincapié ME (2008). TIRAP (MAL) S180L polymorphism is a common protective factor against developing tuberculosis and systemic lupus erythematosus.. Infect Genet Evol.

[28] Law K, Weiden M, Harkin T, Tchou-Wong K, Chi C (1996). Increased release of interleukin-1 beta, interleukin-6, and tumor necrosis factor-alpha by bronchoalveolar cells lavaged from involved sites in pulmonary tuberculosis.. Am J Respir Crit Care Med.

[29] Tsao TC, Hong J, Li LF, Hsieh MJ, Liao SK (2000). Imbalances between tumor necrosis factor-alpha and its soluble receptor forms, and interleukin-1beta and interleukin-1 receptor antagonist in BAL fluid of cavitary pulmonary tuberculosis.. Chest.

[30] Ladel CH, Blum C, Dreher A, Reifenberg K, Kopf M (1997). Lethal tuberculosis in interleukin-6-deficient mutant mice.. Infect Immun.

[31] Juffermans NP, Florquin S, Camoglio L, Verbon A, Kolk AH (2000). Interleukin-1 signaling is essential for host defense during murine pulmonary tuberculosis.. J Infect Dis.

[32] Fremond CM, Togbe D, Doz E, Rose S, Vasseur V (2007). IL-1 receptor-mediated signal is an essential component of MyD88-dependent innate response to Mycobacterium tuberculosis infection.. J Immunol.

[33] Keller C, Lauber J, Blumenthal A, Buer J, Ehlers S (2004). Resistance and susceptibility to tuberculosis analysed at the transcriptome level: lessons from mouse macrophages.. Tuberculosis.

[34] Conner JR, Smirnova II, Poltorak A (2008). Forward genetic analysis of Toll-like receptor responses in wild-derived mice reveals a novel antiinflammatory role for IRAK1BP1.. J Exp Med.

[35] Schwertschlag US, Trepicchio WL, Dykstra KH, Keith JC, Turner KJ (1999). Hematopoietic, immunomodulatory and epithelial effects of interleukin-11.. Leukemia.

[36] Curti A, Ratta M, Corinti S, Girolomoni G, Ricci F (2001). Interleukin-11 induces Th2 polarization of human CD4(+) T cells.. Blood.

[37] Cairo MS, Plunkett JM, Schendel P, van de Ven C (1994). The combined effects of interleukin-11, stem cell factor, and granulocyte colony-stimulating factor on newborn rat hematopoiesis: significant enhancement of the absolute neutrophil count.. Exp Hematol.

[38] Musashi M, Yang YC, Paul SR, Clark SC, Sudo T (1991). Direct and synergistic effects of interleukin 11 on murine hemopoiesis in culture.. Proc Natl Acad Sci U S A.

[39] Trepicchio WL, Bozza M, Pedneault G, Dorner AJ (1996). Recombinant human IL-11 attenuates the inflammatory response through down-regulation of proinflammatory cytokine release and nitric oxide production.. J Immunol.

[40] Lgssiar A, Hassan M, Schott-Ohly P, Friesen N, Nicoletti F (2004). Interleukin-11 inhibits NF-kappaB and AP-1 activation in islets and prevents diabetes induced with streptozotocin in mice.. Exp Biol Med.

[41] Chen Q, Rabach L, Noble P, Zheng T, Lee CG (2005). IL-11 Receptor in the Pathogenesis of IL-13-Induced Inflammation and Remodeling.. J Immunol.

[42] Palmqvist P, Lundberg P, Lundgren I, Hänström L, Lerner UH (2008). IL-1beta and TNF-alpha regulate IL-6-type cytokines in gingival fibroblasts.. J Dent Res.

[43] White CA, Dimitriadis E, Sharkey AM, Stoikos CJ, Salamonsen LA (2007). Interleukin 1 beta is induced by interleukin 11 during decidualization of human endometrial stromal cells, but is not released in a bioactive form.. J Reprod Immunol.

[44] Mohan VP, Scanga CA, Yu K, Scott HM, Tanaka KE (2001). Effects of tumor necrosis factor alpha on host immune response in chronic persistent tuberculosis: possible role for limiting pathology.. Infect Immun.

[45] Keller C, Hoffmann R, Lang R, Brandau S, Hermann C (2006). Genetically determined susceptibility to tuberculosis in mice causally involves accelerated and enhanced recruitment of granulocytes.. Infect Immun.

[46] Beisiegel M, Kursar M, Koch M, Loddenkemper C, Kuhlmann S (2009). Combination of host susceptibility and virulence of Mycobacterium tuberculosis determines dual role of nitric oxide in the protection and control of inflammation.. J Infect Dis.

[47] Biermann H, Pietzn B, Dreier R, Schmid KW, Sorg C (1999). Murine leukocytes with ring-shaped nuclei include granulocytes, monocytes, and their precursors.. J Leukoc Biol.

[48] Sasmono RT, Ehrnsperger A, Cronau AL, Ravasi T, Kandane R (2007). Mouse neutrophilic granulocytes express mRNA encoding the macrophage colony-stimulating factor receptor (CSF-1R) as well as many other macrophage-specific transcripts and can transdifferentiate into macrophages in vitro in response to CSF-1.. J Leukocyte Biol.

[49] Kapina MA, Shepelkova GS, Mischenko VV, Sayles P, Bogacheva P (2007). CD27low CD4 T Lymphocytes That Accumulate in the Mouse Lungs during Mycobacterial Infection Differentiate from CD27high Precursors In Situ, Produce IFN-gamma, and Protect the Host against Tuberculosis Infection.. J Immunol.

[50] Bates DM, Watts DG (1988). Nonlinear regression analysis and its applications.

[51] Burnham KP, Anderson DR (2002). Model selection and multimodel inference: a practical information – theoretic approach..

[52] Efron B, Tibshirani R (1993). An introduction to the bootstrap..

