# Supplementary material for: In Mice, Tuberculosis Progression Is Associated with Intensive Inflammatory Response and the Accumulation of Gr-1dim Cells in the Lungs
Source: PLoS One. 2010 May 4;5(5):e10469. doi: 10.1371/journal.pone.0010469 (PMC2864263; doi:10.1371/journal.pone.0010469)
Supplement: File S1 — Removing severely wasting mice from the analysis. (0.02 MB DOC) [file pone.0010469.s001.doc]

***Removing severely wasting mice from the analysis.*** As shown in the paper, simple correlation analysis of the weight loss and a single factor, performed in female mice, revealed the cytokines IL-1β, IL-6, IL-11, the chemokines CCL3, CCL4, CXCL2, and the metalloproteinase MMP-8 as correlates of TB progression. To identify which of these factors were the main correlates of TB progression we decided to perform multiple regression analysis. Before commencing the multiple regression analysis, we needed to verify that the analyzed female mice contained no out-groups (as based on the weight loss). By plotting weight loss vs. mycobacterial load and performing hierarchical clustering, we found that mice that wasted by more than 20% were outliers and had much higher mycobacterial loads and weight loss than all other mice. These severely wasting female mice contributed significantly to the correlation between the weight loss and TB load (p-value=3,8x10^-3^, Spearman analysis; p-value = 3,5x10^-5^, Pearson analysis), since their removal made the correlation between mycobacterial load and weight loss statistically non-significant (p-value = 0,31 and 0,24 for Spearman and Person correlation analysis, respectively, Figure S1).
