# Supplementary material for: In Mice, Tuberculosis Progression Is Associated with Intensive Inflammatory Response and the Accumulation of Gr-1dim Cells in the Lungs
Source: PLoS One. 2010 May 4;5(5):e10469. doi: 10.1371/journal.pone.0010469 (PMC2864263; doi:10.1371/journal.pone.0010469)
Supplement: File S2 — Multiple regression analysis and F-tests. (0.02 MB DOC) [file pone.0010469.s002.doc]

***Multiple regression analysis and F-tests.*** The analysis was performed using a cutoff for the weight loss of 20% (total n=68 mice). When all factors were taken into account in the regression analysis (Table S1, full model) only TNF-α contributed significantly (but weakly) to the weight loss. This suggested that expression of different factors in different mice was not well correlated. Next we asked whether all the factors (shown in Table S1) taken together explained the data better than any single factor (IL-11, Mycobacterial load etc). For that we performed linear regression analysis (weight loss vs. single factor) and compared the results of the fitting with the full model. The F-test showed that IL-11, IL-1β, and MMP-8 by themselves explained the data well, and that other factors were not required to explain the weight loss.
