# Supplementary material for: In Mice, Tuberculosis Progression Is Associated with Intensive Inflammatory Response and the Accumulation of Gr-1dim Cells in the Lungs
Source: PLoS One. 2010 May 4;5(5):e10469. doi: 10.1371/journal.pone.0010469 (PMC2864263; doi:10.1371/journal.pone.0010469)
Supplement: File S3 — Selecting a minimal model. Akaike and Bayesian Information Criterion. (0.02 MB DOC) [file pone.0010469.s003.doc]

***Selecting a minimal model. Akaike and Bayesian Information Criterion.*** A second approach is to use Akaike or Bayesian Information Criterion (AIC or BIC) to select the best model [51]. One disadvantage of this approach is that BIC/AIC cannot be used to test statistical hypothesis and therefore, one cannot claim that the best selected model is indeed statistically superior than other models. In R, selection of the best linear model using BIC/AIC can be done with the routine step (Table S1, the minimal model; also see below).

The difference in AIC between the minimal and the full model was relatively small, Δ = 1.3 (similar results for BIC, not shown), suggesting that the minimal model with only IL-11 as the predictor of weight loss described the data with a reasonable quality.
