# Supplementary material for: In Mice, Tuberculosis Progression Is Associated with Intensive Inflammatory Response and the Accumulation of Gr-1dim Cells in the Lungs
Source: PLoS One. 2010 May 4;5(5):e10469. doi: 10.1371/journal.pone.0010469 (PMC2864263; doi:10.1371/journal.pone.0010469)
Supplement: File S4 — Bootstrapping to account for variability. (0.02 MB DOC) [file pone.0010469.s004.doc]

***Bootstrapping to account for variability.*** Finally, by bootstrapping the data for moderately wasting and gaining mice [52], we have investigated which minimal model explains the best data on weight loss. Specifically, we re-sampled the data with replacement to create a pseudo dataset, and then used routine *step* in R to determine best minimal model. In 1000 bootstraps, IL-11 came as a best single predictor in 10.7% cases.

Distribution of the weight loss in all female mice was clearly non-normal, and therefore, an alternative way to select for severely wasted mice (in the initial subset of 75 mice) was to look for weight outliers in the normal Q-Q plots. Simple analysis showed that mice with weight loss of more than 12% could be the outliers since their removal made the distribution of weight loss normal (results not shown). We therefore performed a similar regression analysis of the data from mice that lost less than 12% of the initial weight (n=64). This analysis confirmed an important role for IL-11 and also IL-1β as the best single predictors of the weight loss. In bootstrapping the data, IL-1β and IL-11 came as the best single predictors of the weight loss in 13.2% and 6.2% cases, respectively. Also, IL-1β could explain the data on weight loss with a similar quality as the model with three factors, IL-11, IL-1β, TNF-α, selected by the routine *step* (F-test for nested models, p = 0.059). Therefore, in mice that lost less than 12% of the initial weight, IL-1β and to a lesser extent IL-11, were the best factors explaining weight loss. Using bootstrap analysis, we frequently found TNF-α to be part of the model, but never alone. If we performed a multiple regression analysis including TNF-α, we saw that, contrary to IL-1β and IL-11, it had small negative correlation with weight loss. When only simple correlation was performed, we observed that TNF-α correlated directly, but only weakly with weight loss. When other factors, especially IL-1β and IL-11 were taken into account, TNF-α turned out to have a small protective effect.
