# Supplementary figures and images for: In Mice, Tuberculosis Progression Is Associated with Intensive Inflammatory Response and the Accumulation of Gr-1dim Cells in the Lungs
Source: PLoS One. 2010 May 4;5(5):e10469. doi: 10.1371/journal.pone.0010469 (PMC2864263; doi:10.1371/journal.pone.0010469)

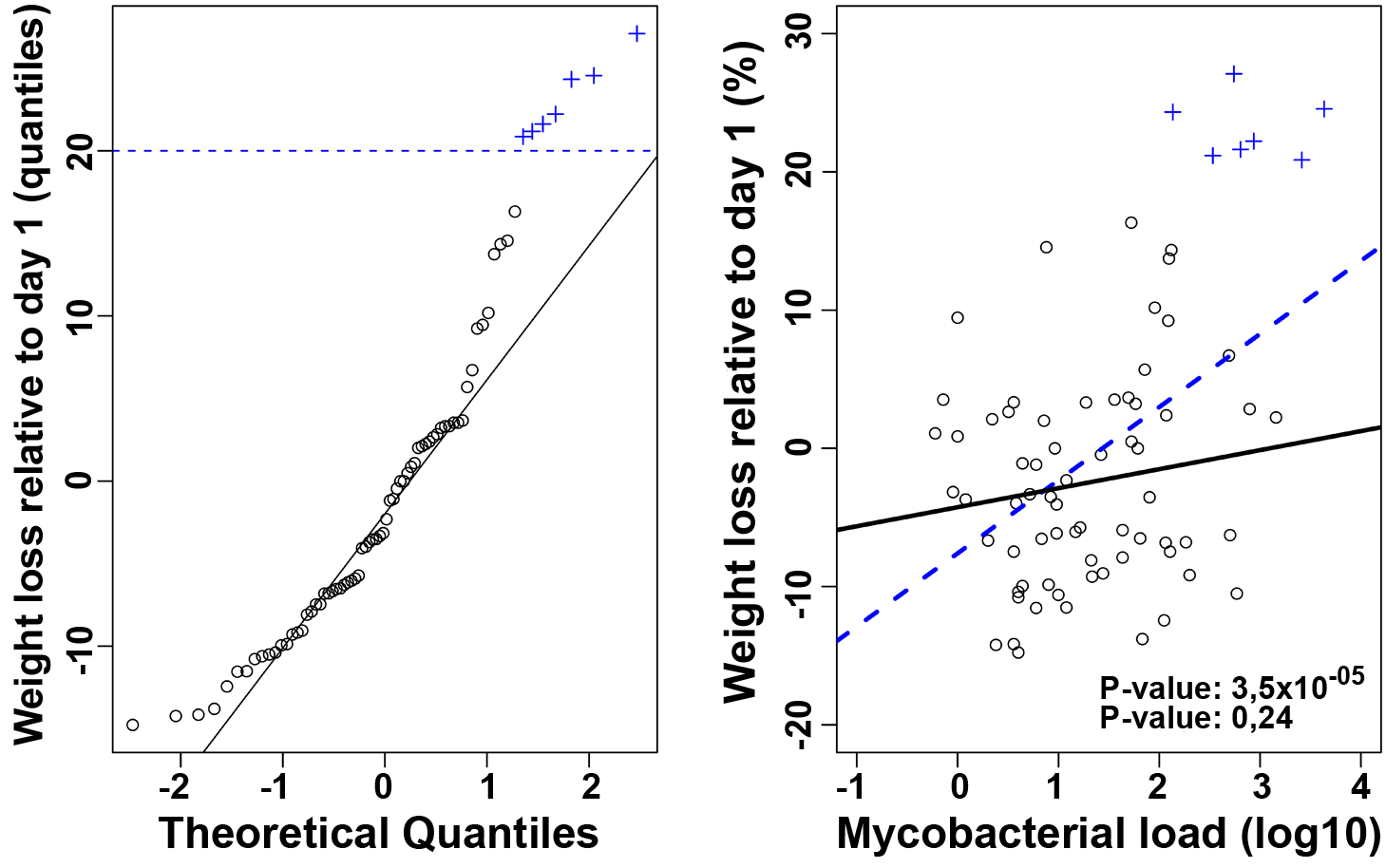

Supplement: Figure S1 — Correlation between disease progression (as determined by the weight loss at day 24 post-infection) and Mtb load for female mice. Dashes blue line, the prediction of the linear regression between weight loss and Mtb load when all mice are included in the analysis; solid black line, the same when severely wasting mice (shown by blue crosses) are excluded from the analysis. (4.03 MB TIF) [file pone.0010469.s005.tif]
