# Supplementary material for: In Mice, Tuberculosis Progression Is Associated with Intensive Inflammatory Response and the Accumulation of Gr-1dim Cells in the Lungs
Source: PLoS One. 2010 May 4;5(5):e10469. doi: 10.1371/journal.pone.0010469 (PMC2864263; doi:10.1371/journal.pone.0010469)
Supplement: Table S1 — Results of the linear regression analysis of the weight loss and all factors in gaining and moderately wasting mice. (0.04 MB DOC) [file pone.0010469.s006.doc]

***Table S1. Results of the linear*** regression analysis of the weight loss and all factors in gaining and moderately wasting mice.

| Factors | Estimate | Std. Error | t value | Pr(>t) |
| --- | --- | --- | --- | --- |
| (Intercept)^a^ | 0.6574 | 7.8274 | 0.08 | 0.9334 |
| Lung infiltration ^a^ | 0.0077 | 0.0770 | 0.1 | 0.9207 |
| Mycobacterial ^a^ load ^a^ | -1.7960 | 1.4995 | -1.2 | 0.2360 |
| IL-1β ^a^ | 4.6318 | 4.4452 | 1.04 | 0.3018 |
| IL-11 ^a^ | 2.0600 | 2.0775 | 0.99 | 0.3256 |
| CCL3 ^a^ | -1.0725 | 5.3004 | -0.2 | 0.8404 |
| CXCL2 ^a^ | 5.8001 | 4.4302 | 1.31 | 0.1957 |
| MMP-8 ^a^ | 1.8179 | 2.8652 | 0.63 | 0.5283 |
| TNF-α ^a^ | **-12.8726** | **5.5539** | **-2.32** | **0.0241** |
| iNOS ^a^ | 3.6346 | 4.2582 | 0.85 | 0.3969 |
| (Intercept) ^b^ | 12.5205 | 3.6861 | 3.4 | 0.0012 |
| IL-11 ^b^ | **4.6855** | **1.1190** | **4.19** | **0.0001** |

^a^, The full model. All indicated factors were taken into account in the regression analysis. Note that only TNF-α correlated weakly with the weight loss.

^b^, The minimal model. The initial model included IL-11, IL-1β, and MMP-8 and was fitted to the data using linear regression in R. The minimal model that explained the data with the best quality was determined using a routine *step* in R with the Baysian Information Criterion, BIC [51]. If we used Akaike Information Criterion (AIC, [51]), then both IL-11 and IL-1β were selected as best predictors but only estimate for the slope of IL-11 was statistically different from zero with p= 0,029 (results not shown).
